# Meningioma of the Fourth Ventricle of the Brain: A Case Report

**DOI:** 10.7759/cureus.55011

**Published:** 2024-02-27

**Authors:** Shilong Sun, Houjie Zhou

**Affiliations:** 1 Neurosurgery, Peking University Shenzhen Hospital, Shenzhen, CHN

**Keywords:** lateral ventricular puncture drain, benign tumor, fibrous meningioma, posterior median approach, fourth ventricular meningioma

## Abstract

Meningiomas constitute a significant proportion of primary intracranial tumors; however, their occurrence within the brain’s ventricles is exceptionally uncommon. This report details the case of a 24-year-old man who presented with six months of diplopia. Diagnostic imaging revealed a mass in the fourth ventricle, causing obstructive hydrocephalus. The patient underwent a lateral ventricular puncture for drainage, followed by surgical removal of the tumor. Histopathological examination identified the mass as a fibrous World Health Organization grade 1 meningioma. Symptoms were significantly relieved after surgery, and follow-up imaging results after three months showed that the patient had recovered well from surgery, with no residual lesions and no recurrence of the tumor. Intraventricular meningiomas often pose diagnostic challenges because of their resemblance to more prevalent intracranial lesions on imaging studies such as choroid plexus papillomas. This case underscores the critical role of histopathological analysis in establishing a definitive diagnosis. Surgical excision is the primary treatment strategy for intraventricular meningiomas, generally resulting in positive outcomes.

## Introduction

Meningiomas are the most common intracranial nonmalignant tumors, with an incidence of 37.6% [1]. In contrast, meningiomas involving the interventricular spaces are rare, accounting for only 0.5% to 5% of intracranial meningiomas [2]. Lateral ventricular meningiomas are the most prevalent among ventricular meningiomas, with over 80% of such tumors occurring in the lateral ventricles [3]. In our patients, the tumors were located in the fourth ventricle with an incidence of approximately 0.08%. The clinical features, imaging characteristics, and long-term prognosis of these tumors are different from those of meningiomas in other parts of the brain, and they are often confused with other tumors in the fourth ventricle, thus posing a diagnostic challenge. Surgery is most often performed using a suboccipital median craniotomy to remove fourth ventricle tumors. Resection of fourth ventricle tumors is challenging due to the proximity of vital structures. In order to protect neurological function, it is preferable to use a distal incision when removing the tumor. We report this case to deepen the understanding of fourth ventricular meningiomas (FVMs).

## Case presentation

A 24-year-old man presented to the neurosurgery clinic with three weeks of diplopia. He had no headache, nausea, vomiting, or other symptoms, nor did he experience aura or seizures. Additionally, the patient had no history of head trauma or falls. There was no history of radiation therapy to the head or face, and no history of smoking or alcohol consumption. There was also no family history of associated tumors. He also did not report any memory loss or personality changes.

Examination revealed that the patient had a slightly impaired heel-knee-tibia test, rotation test, and finger-nose test. His tongue was properly aligned, his pupils were symmetrical and round, and his light reflex was responsive. Additionally, there was no family history of tumors.

**Figure 1:**
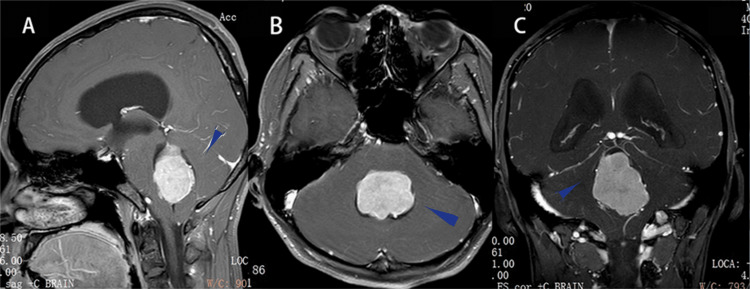
Preoperative magnetic resonance imaging T1 enhancement scan (A-C) The arrow is pointing toward the lesion.

Blood tests from samples drawn on admission showed no hormonal imbalance. An ophthalmology consultation led to the diagnosis of incomplete paralysis of the abducens nerve bilaterally, with visual acuity of 0.5 in the right and left eyes. Subsequently, he underwent intensified cranial magnetic resonance imaging (MRI), which showed an ill-defined heterogeneous lesion with supratentorial hydrocephalus at the fourth ventricle (Figures 1A-1C), initially considered to be a papilloma of the choroid plexus.

After completing the preoperative evaluation, the patient was subjected to general anesthesia. He was first placed in the supine position and underwent external drainage by the puncture of the frontal horn of the right ventricle. Then, he was placed in the prone position with his head fixed in a three-pin headframe. Intraoperative electrophysiologic monitoring was used to assess brainstem function; this was performed using the posterior median approach.

The tumor, located in the fourth cerebral ventricle, was extruding the anterior part of the brain and was demarcated from the surrounding brain tissues with an abundant blood supply. The tumor was carefully separated and resected in pieces along the border, with attention paid to protecting the brain and peripheral neurovascular vessels. First, a portion was removed postoperatively and sent for a frozen section, which showed a meningioma. Then, the tumor was completely resected under the microscope.

**Figure 2:**
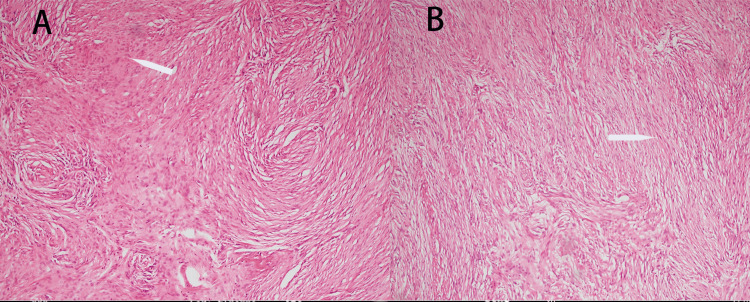
(A) The white arrows point to collagen fibers (magnification ×10); (B) The white arrow points to a mat-like structure (magnification ×10)

The operation proceeded smoothly, with bleeding of about 200 mL and no need for blood transfusion. The patient’s vital signs remained stable throughout the operation. Afterward, he was returned to the neurosurgical intensive care unit with an endotracheal tube in place. Per the standard procedure, the resected tumor tissue was sent for pathological analysis (Figures 2A-2B).

**Figure 3:**
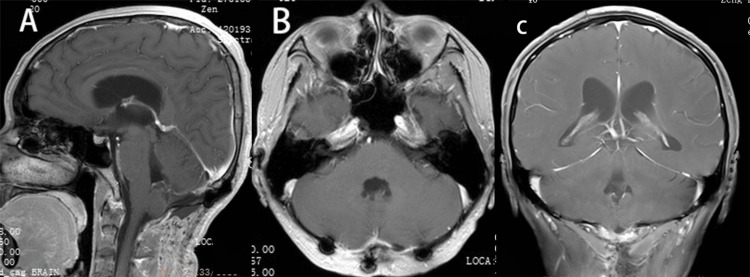
(A-C) This scan was performed after discharge from the hospital and no significant enhancing lesions were found.

A postoperative MRI scan showed no signs of residual lesions. Additionally, no intracranial hemorrhage, regional infarcts, or abnormally enhancing lesions were detected, and the patient’s symptoms improved significantly (Figures 3A-3C).

The patient was advised to undergo regular follow-up after discharge from the hospital. The frequency is three months, six months, and one year.

## Discussion

FVMs arise from the choroid plexus without dural attachment, and therefore, neither dural tail sign on MRI nor tumor staining from the meningeal branches are usually observed [4]. Choroid plexus papilloma is a difficult lesion to differentiate from meningioma; however, choroid plexus has a higher amount of vascularity, with globular formations causing irregular contours, frequent calcifications, and hydrocephalus. It is extremely difficult to differentiate them from choroid plexus papillomas [5]; however, the choroid plexus is typically associated with more blood vessels and globular structures leading to irregular contours, frequent calcifications, and hydrocephalus. The symptoms of FVMs are diverse [6]. Tumor obstruction of cerebrospinal fluid pathways can lead to increased intracranial pressure due to obstructive hydrocephalus [7], and some cases present with focal neurological deficits such as diplopia [8], long fasciculus sign [9], and cerebellar sign [10]. The age distribution of the patients ranged from 28 to 66 years (average age, 46.5 years). There was a slight male predominance. Of these, 82.8% were World Health Organization (WHO) grade I tumors and 17.2% were WHO grade II tumors [11].

The primary treatment for meningiomas is surgery; meningiomas located in the fourth ventricle may cause supratentorial hydrocephalus, and lateral ventricular puncture and drainage may be chosen to relieve obstructive symptoms. For long-term relief, total resection should be performed using a suboccipital approach [11]. Intraoperative neurophysiologic monitoring is routinely employed to safeguard neurological function. After total tumor resection, the obstruction in the fourth ventricle is released, eliminating the need for a permanent ventriculoperitoneal shunt.

For the treatment of grade 1 meningiomas, surgery or radiosurgery is usually sufficient, with adjuvant radiation therapy considered only if the residual tumor enlarges [2]. For grades 2-3 meningiomas, a combination of surgery and adjuvant radiation therapy is recommended [12]. According to the European Society for Neuro-Oncology guidelines, fractionated radiotherapy is not required for patients who undergo total resection but is recommended for those who undergo subtotal or partial resection. Regular follow-up is essential to prevent possible irreversible neurological deficits and to determine the optimal timing for potential intervention or re-intervention. The recurrent rate of FVMs is about 6.8%, a little higher than the rate of intraventricular meningiomas (5.3%) [2].

## Conclusions

FVMs are rare tumors that cannot be easily distinguished from other tumors of the fourth ventricle preoperatively. In this paper, we present a case of FVM in a young patient. These tumors exhibit distinct characteristics in terms of age of onset, sex ratio, and histologic type. The recommended treatment for FVMs is surgery through a distal approach with suboccipital craniectomy to achieve total resection. The prognosis for FVMs is relatively favorable, with fewer postoperative complications and a higher rate of total resection compared with other fourth ventricle tumors.

This report may contribute to a deeper understanding of this rare clinical entity. More cases are needed in the future to continue monitoring and research in the field of FVMs. This is quite necessary.
